# Differential Expression of Long Noncoding RNA in Primary and Recurrent Nasopharyngeal Carcinoma

**DOI:** 10.1155/2014/404567

**Published:** 2014-04-14

**Authors:** Wei Gao, Jimmy Yu-Wai Chan, Thian-Sze Wong

**Affiliations:** Department of Surgery, LKS Faculty of Medicine, The University of Hong Kong, Queen Mary Hospital, 102 Pokfulam Road, Hong Kong, Hong Kong

## Abstract

*Background*. Recent studies suggested that non-protein-coding genes are implicated in the tumorigenic process of nasopharyngeal carcinoma (NPC). In the present study, we aimed to identify the differentially expressed long noncoding RNA (lncRNA) using data available in the public domain. * Methods*. Microarray data set GSE12452 was reannotated with ncFANs. Real-time quantitative PCR was used to quantify and validate the identified lncRNAs in NPC. * Results*. In primary NPC, upregulation of lnc-C22orf32-1, lnc-AL355149.1-1, and lnc-ZNF674-1 was observed. High levels of lnc-C22orf32-1 and lnc-AL355149.1-1 were significantly associated with the male patients. In addition, increased expression of lnc-C22orf32-1 and lnc-ZNF674-1 was associated with advanced tumor stages. Recurrent NPC displayed a distinctive lncRNA expression pattern. lnc-BCL2L11-3 was significantly increased in the recurrent NPC tissues. In addition, significant reduction of lnc-AL355149.1-1 and lnc-ZNF674-1 was observed in the recurrent NPC tissues. * Conclusions*. Our results demonstrated that it is feasible to identify the differentially expressed lncRNA in the microarray dataset by functional reannotation. The association of lncRNA with gender and tumor size implicated that lncRNA possibly plays a part in the pathogenesis of primary NPC. Further, the distinctive lncRNA identified in the recurrent NPC may reveal a distinctive development mechanism underlying tumor recurrence.

## 1. Introduction

Undifferentiated nasopharyngeal carcinoma (NPC) is prevalent in specific geographic localities including Southern China and East Asia, Southeast Asia, Greenland (native), Canada (Northwest Territories), and Alaska. Undifferentiated NPC is characterized by its close association with the virology of Epstein-Barr virus, a gamma-herpes DNA virus that infects more than 90% of world's population and is found in nearly all undifferentiated NPC cells [1]. According to the World Health Organization classification (WHO 2005), NPC could be classified based on the cancer differentiation status into type 1 keratinising squamous cell carcinoma and type 2 nonkeratinising carcinoma (differentiated and undifferentiated NPC). In endemic regions, the major histological type is type 2 NPC [2]. Early diagnosis of NPC is difficult as the cancer is developed from the epithelial lining around the ostium of the Eustachian tube in the lateral wall of the nasopharynx with close proximity to the skull [3]. Later diagnosis will usually lead to unsatisfactory treatment results and high incidence of local regional recurrence [4].

lncRNAs are non-protein-coding transcripts transcribed by either RNA Pol I, RNA Pol II, or RNA Pol III. Different from the protein-coding genes, lncRNA has distinct biological function in maintaining the cellular homeostasis [5]. Prensner and Chinnaiyan summarized our current knowledge on lncRNA in the pathogenesis and progression of human malignancies [6]. At present, we know that long or large intergenic ncRNAs, transcribed ultraconserved regions, pseudogenes, enhancer RNAs, antisense RNAs, and long stress-induced noncoding transcribes are involved in the development and tumorigenesis of cancers. The existence of these noncoding RNAs used to be regarded as transcriptional byproducts with no significant functions. Over the past decade, however, increasing evidence suggested that lncRNA could function as epigenetic regulators, modulators of gene expression, and protein degradation machinery as well as regulators of organelle biogenesis and subcellular trafficking [7]. Hence, further understanding of the aberrant expression pattern of lncRNA in cancers is essential.

Study on lnRNA deregulation in undifferentiated NPC remains raw. Recently, with the use of candidate gene approach, it was identified that HOX antisense intergenic RNA (HOTAIR), one of the first described lncRNAs transcribed in the HOXC locus at 12q13.13, was suggested to play a part in NPC. HOTAIR was implicated in cancer progression and metastasis. In primary NPC, high HOTAIR expression was specific to the primary NPC and was absent in the normal nasopharyngeal epithelia. HOTAIR expression level was linked with the tumor size, nodal status, and distant metastasis. Further, patients with high HOTAIR levels will have poor clinical outcome with tumor recurrence and distant metastasis [8, 9].

High-throughput microarray was originally designed to detect and quantify the protein-coding genes. However, it is now noted that many of the probes on the microarray also match with the sequence of lncRNA. By rematching and reannotation of the existing microarray datasets, the results obtained from the microarray experiments could be used for lncRNA analysis concurrently. For example, with the use of Affymetrix U133A and B microarrays, it has been demonstrated that the microarray data can be reannotated for lncRNA expression analysis [10]. Here, we explored the differential lncRNA expression patterns in nasopharyngeal carcinoma (NPC) based on the high-throughput NPC dataset in the public domain using the functional reannotation service provided by noncoding RNA Function ANnotation server (ncFANs) [11]. The identified lncRNAs were validated in both primary and recurrent NPC samples.

## 2. Materials and Methods

### 2.1. Dataset

Microarray data set GSE12452 containing 41 microarray data (http://www.ncbi.nlm.nih.gov/geo/query/acc.cgi?acc=GSE12452) was collected from Gene Expression Omnibus (GEO). The dataset contains 31 NPC and 10 normal nasopharyngeal tissue samples examined with Human Genome U133 Plus 2.0 Array (HG-U133 Plus 2) from Affymetrix [12]. The tissues were confirmed histologically before the microarray experiment. Tumor tissue was defined by the positive stain for both cytokeratins and EBV EBERs using immunohistochemical staining. All the samples were processed by laser-captured microdissection to enrich the epithelial cells before total RNA extraction.

### 2.2. Patient Samples

Undifferentiated NPC tissue samples were obtained from Department of Surgery, The University of Hong Kong, Queen Mary Hospital, Hong Kong. Written consent of tissue donation for research purposes was obtained from patients before tissue collection. The protocol was approved by the IRB of the hospital (reference number UW 10-142). Tissue samples were stored at liquid nitrogen for transportation and stored at −70°C before use. In total, nasopharyngeal tissue samples were collected from 42 patients with primary NPC, 33 patients with recurrent NPC, and 29 noncancer volunteers. The 42 patients with primary NPC included 32 male and 10 female, with age ranging from 12 to 75 years. The 33 recurrent NPC patients included 24 male and 9 female with age ranging from 29 to 75 years. The 29 healthy volunteers included 20 male and 9 female, with ages ranging from 6 to 81 years.

### 2.3. Reannotation and Selection of Differential Expressed lncRNA

The microarray data was preprocessed with the preprocessing program and reannotated with the Affymetrix CDF file from the web server of ncFANs (noncoding RNA Function ANnotation server). The data was normalized by MAS5.0 before lncRNAs annotation. Transcripts with length <200 were excluded [11]. Differentially expressed lncRNAs were selected using Student's *t*-test using *P* value below 0.05 as cutoff.

### 2.4. RNA Extraction and Real-Time Quantitative RT-PCR Validation of the Microarray Results

Total RNA was extracted with TRIZOL reagent (Invitrogen) following the manufacturer's protocol. The cDNA product was generated using cDNA conversion kit (Invitrogen). Primers were designed with Primer3 provided by the Universal ProbeLibrary Assay Design Center (http://www.roche-applied-science.com/). The LNA-labelled probe was obtained from the Universal ProbeLibrary (UPL). The lncRNA transcript levels were quantified on LightCycler 480 (Roche Applied Science) and normalized with the GAPDH levels by 2^−ΔCt^ method. All reactions were done in triplicate. Primer and probe sequences were shown in Table 1.

### 2.5. Statistical Analysis

Statistical analysis was performed with SPSS V16.0 (SPSS, Chicago, IL). The difference between primary NPC and controls was calculated using the Mann-Whitney *U* test. The statistical difference between recurrent NPC and paired normal tissues was examined using Wilcoxon Signed Rank test. All the tests were two-sided. *P* value <0.05 was considered statistically significant.

## 3. Results

### 3.1. Functional Annotation of lncRNA with ncFANs

The differentially expressed lncRNAs identified were listed in Table 2. Five lncRNAs were found to be differentially expressed in the nasopharyngeal carcinoma tissues in comparison with the normal nasopharyngeal epithelial tissues in the microarray data. The lncRNAs identified were lnc-C22orf32-1, lnc-TLR4-1, lnc-BCL2L11-3, lnc-AL355149.1-1, and lnc-ZNF674-1.

### 3.2. lncRNA Validation on Primary NPC Tissues and Normal Nasopharyngeal Epithelia

**Figure 1:**
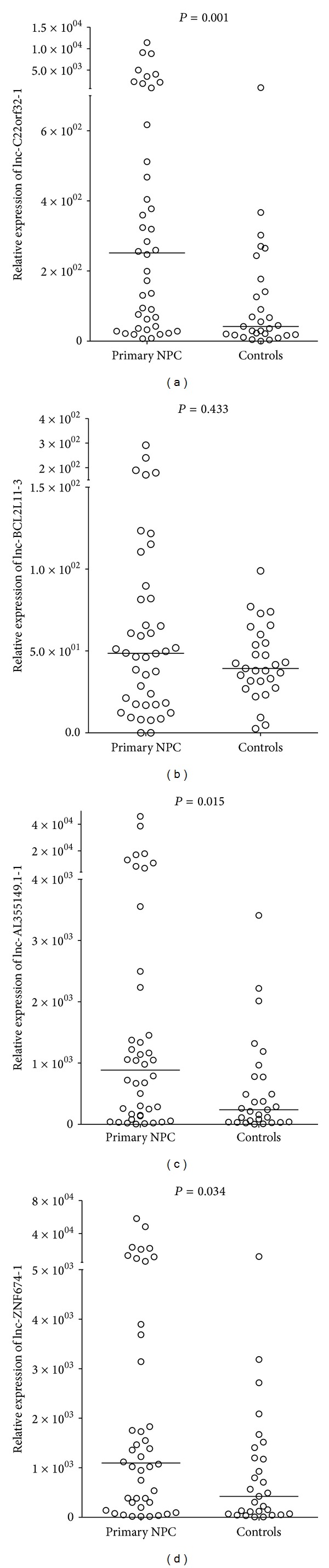
Relative expression levels of lncRNAs in tissue samples from patients with primary NPC and healthy controls. The relative expression levels were normalized to* GAPDH* by qPCR analysis. The difference between primary NPC and controls was calculated using the Mann-Whitney *U* test.

To validate the identified lncRNA, we performed real-time quantitative analysis on the candidate lncRNA in our cohort. First, we examined the fold changes in the primary nasopharyngeal carcinoma and normal nasopharyngeal tissues (Figure 1). Among the 5 candidate lncRNAs, lnc-C22orf32-1, lncTLR4-1, lnc-AL355149.1-1, and lnc-ZNF674-1 demonstrated significant expression difference between the primary NPC and normal nasopharyngeal tissues.

**Figure 2:**
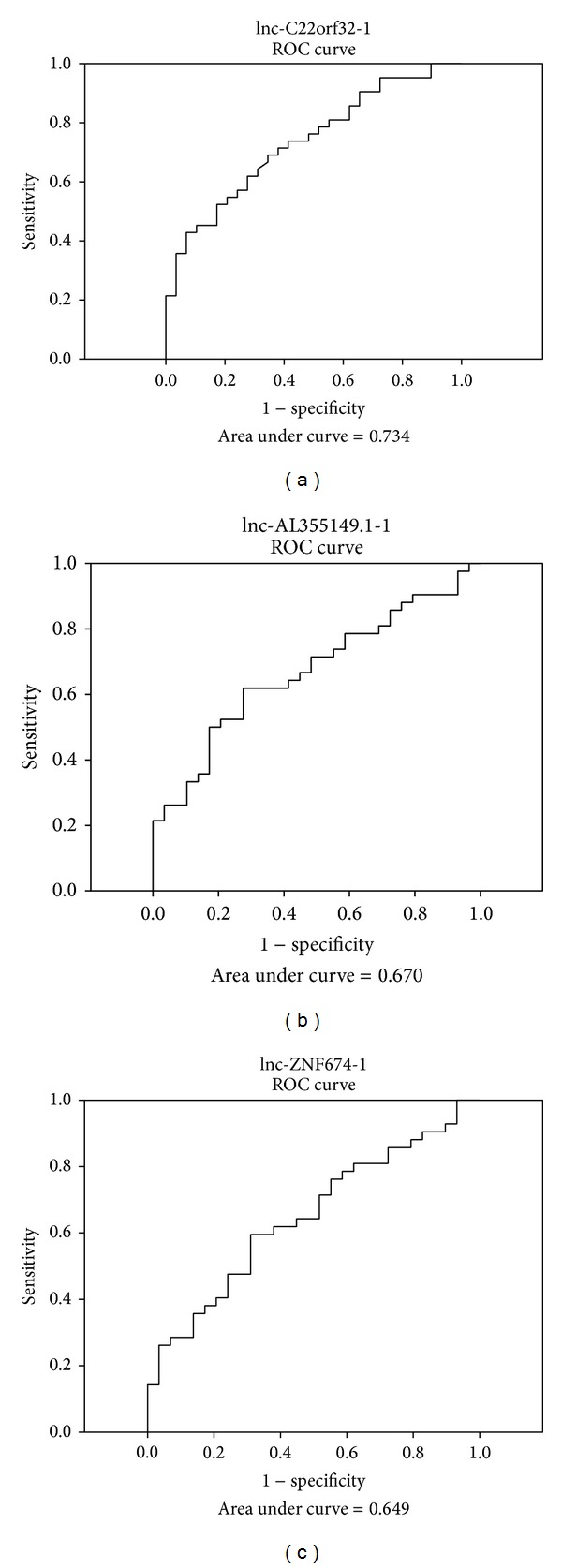
Receiver operating characteristic (ROC) curve analysis of lncRNAs in tissue samples from patients with primary NPC and healthy controls.

Of the 4 lncRNAs, the expression difference of lnc-C22orf32-1 was highly significant (*P* = 0.001, Mann-Whitney *U* test). High lnc-C22orf32-1 was found in the primary NPC tissues in comparison with the normal controls (Figure 1). In ROC analysis, the AUC value of lnc-C22orf32-1 was 0.734 in differentiating NPC from the normal nasopharyngeal tissues (Figure 2); lnc-AL355149.1-1 and lnc-ZNF674-1 were also found to be overexpressed in the primary NPC tissues (*P* = 0.015 and 0.034, respectively, Mann-Whitney *U* test). The 3 lncRNAs above are detectable in all the tissues examined. In contrast, lnc-TLR4-1 was detectable in 21% (9/42) patients with primary NPC and 66% (19/29) healthy controls. In recurrent NPC, lnc-TLR4-1 was detectable in 27% (9/33) tumor tissues and 15% (5/33) paired normal tissues. Low copy number of lnc-TLR4-1 in tissue samples may account for the low detection rate. The expression pattern of lnc-TLR4-1 was not correlated with age or gender (data not shown). Since lnc-TLR4-1 was only expressed in a subset of NPC and normal nasopharyngeal tissues, it was not suitable for study of differential expression.

**Figure 3:**
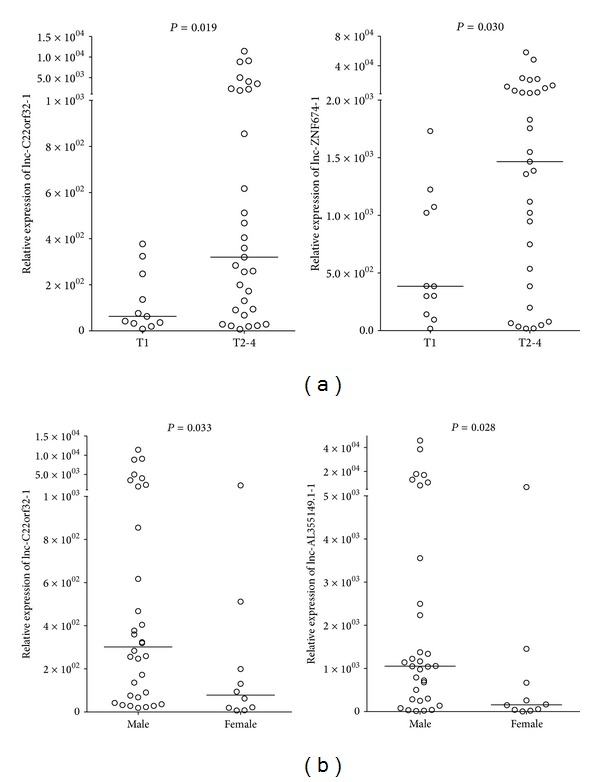
Relative expression levels of lncRNAs in primary NPC patients with different T stage (a) and gender (b). The relative expression levels were normalized to* GAPDH* by qPCR analysis. The difference was calculated using the Mann-Whitney *U* test.

Next, we compared the expression level of the 3 universal expressed lncRNAs with the clinicopathological parameters of the primary NPC patients (Table 3). High levels of lnc-C22orf32-1 and lnc-AL355149.1-1 were significantly associated with the male patients (Figure 3(b)). In addition, increased expression of lnc-C22orf32-1 and lnc-ZNF674-1 was associated with advanced tumor stages (T2–4) in comparison with the early stage (T1) NPC (Figure 3(a)).

### 3.3. Differential lncRNAs Expression in Recurrent NPC and the Paired Normal Epithelia

**Figure 4:**
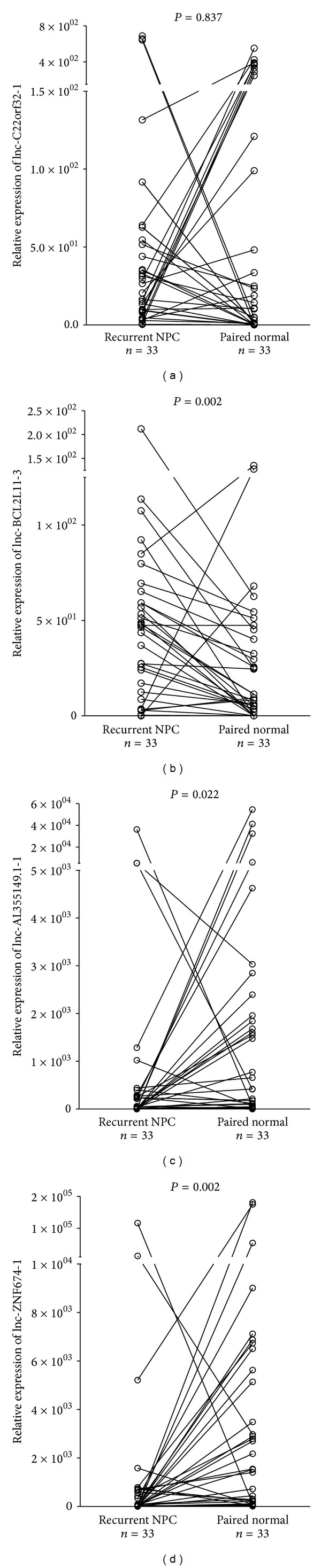
Relative expression levels of lncRNAs in paired tumor and normal tissue samples from patients with recurrent NPC. The relative expression levels were normalized to* GAPDH* by qPCR analysis. The difference between tumor and normal was calculated using the Wilcoxon Signed Rank test.

We compared the lncRNA expression patterns with the paired normal tissues obtained from the same recurrent NPC patients (Figure 4). lnc-BCL2L11-3 was found to be upregulated in the recurrent NPC tissues in comparison with the paired normal tissues (*P* = 0.002, Wilcoxon Signed Rank test). For lnc-AL355149.1-1 and lnc-ZNF674-1, high expression was found in primary NPC. However, significant expression reduction was observed in the recurrent NPC tissues (*P* = 0.022 and 0.002, respectively, Wilcoxon Signed Rank test).

## 4. Discussion

The differential expression of lnc-AL355149.1-1, lnc-C22orf32-1, and lnc-ZNF674-1 observed in NPC might be attributed to epigenetic regulation including microRNA, DNA methylation, and histone modification. As predicted by a bioinformatics tool MirTarget2 (http://mirdb.org/miRDB/index.html), lnc-AL355149.1-1 is targeted by hsa-miR-939, hsa-miR-4283, and hsa-miR-3191-5p; lnc-C22orf32-1 is targeted by hsa-miR-4751; lnc-ZNF674-1 is targeted by hsa-miR-1296, hsa-miR-1292, and hsa-miR-3179. In addition, DNA methylation and histone modification may also contribute to the aberrant expression of these lncRNAs. For example, DNA methylation, H3K4 histone methylation, and H3K9 histone methylation are found in lnc-AL355149.1-1 gene in different human cell lines (lncRNome, http://genome.igib.res.in/lncRNome/).

lncRNAs could exert biological functions by interacting with RNA or protein molecules. An lncRNA, highly upregulated in liver cancer (HULC), could bind to miR-372, thus modulating the expression of miR-372 at posttranscriptional level [13]. lncRNA HOTAIR interacted with polycomb chromatin remodeling complex 2 (PRC2) by its 5′ domain and bind to LSD1/CoREST/REST complex via its 3′ domain, resulting in a specific histone methylation pattern [14]. To explore the possible model by which these differentially expressed lncRNAs exert their biological functions, we tried to predict their binding capacity via database lncRNome. The nucleotides 595–597 of lnc-ZNF674-1 are predicted to be protein binding sites, indicating that it may play a role in NPC by interacting with specific proteins.

Primary NPC is highly sensitive to radiotherapy and the use of concurrent chemoradiotherapy has significantly improved the local control rates of NPC [15]. Nevertheless, local recurrence still appears in approximately 10–30% of patients after primary radiotherapy [16]. Since recurrent NPC is possibly derived from those NPC cells that could escape from radiotherapy, the recurrent NPC might exhibit distinctive mRNA, miRNA, and protein expression patterns in comparison with primary NPC. For instance, the expression levels of miR-98, TR-*α*2 mRNA, multidrug resistance 1 (MDR-1) protein, and glutathione-S-transferase-*π* (GST-*π*) protein were different between recurrent NPC and primary NPC [17, 18]. Likewise, in this study, we also observed a diverse lncRNA expression pattern between recurrent NPC and primary NPC. For example, the aberrant expression of lnc-BCL2L11-3 was only observed in recurrent NPC but not in primary NPC, suggesting that lncRNAs might play distinct roles in the initiation and progression of recurrent NPC and primary NPC.

According to Global Cancer Statistics [19], NPC is a male-dominant disease with incidence rates usually 2 to 3 times lower in females. In our cohort, the ratio between males and females is about 3. Therefore, one of the limitations of this study is the small sample size of females. Another concern about our patient samples is the large age range. To investigate whether the large age range would affect the expression level of lncRNAs, we divided our patients into two groups according to various cutoff values of age. There are no significant differences in the expression of lncRNAs between the two groups of patients divided by varied cutoff values of age.

The differential lncRNA expression patterns could be represented by the microarray probsets, which were originally intended to map the somatic gene expression level. This approach has proven successful in identifying the altered lncRNA expression in the nucleus accumbens of heroin abusers. Michelhaugh SK reanalyzed the existing Affymetrix microarray dataset and identified the 5 lncRNAs dysregulated in the heroin abusers [10]. They curated existing RNA databases including RNAdb and H-Invitational to generate a list of lncRNA and later matched them with the probeset on the Affymetrix U133A and U133B chips [10]. Using similar approaches, we demonstrated that the differential lncRNA expression data could possibly be retrieved from the existing microarray dataset for use, to identify candidate lncRNA which is implicated in the pathogenesis of NPC.

## 5. Conclusions

Our results demonstrated that it is feasible to identify the differentially expressed lncRNA in the microarray dataset by functional reannotation. The association of lncRNA with gender and tumor size implicated that lncRNA possibly plays a part in the pathogenesis of primary NPC. Further, the distinctive lncRNA identified in the recurrent NPC may reveal a distinctive development mechanism underlying tumor recurrence. Further studies are warranted to explore the pathological significance of lncRNA in the development of NPC.

## Figures and Tables

**Table 1:** Primer and probe sequences used in quantitative PCR.

Vega gene ID	LNCipedia gene ID	Forward primer (5′-3′)	Reverse primer (5′-3′)	UPL number*
OTTHUMG00000153695	lnc-BCL2L11-3	agcagatgctgtgcctgata	cctttctcgacccagaagc	#67
OTTHUMG00000002490	lnc-AL355149.1-1	gaaaactaggcgtctgggaac	caaacaatgggagcaagtcc	#25
OTTHUMG00000150697	lnc-C22orf32-1	tgctcatcttctgccacagt	agggcagtgatgaggaacc	#38
OTTHUMG00000021416	lnc-ZNF674-1	agcacttggccctaaagaga	aacatactggcccaaacagc	#88
OTTHUMG00000020561	lnc-TLR4-1	ccacacaaatgggcaagaat	gcaaaatcctgaaggttcaaa	#6

*Probe number in the universal probe library (UPL).

**Table 2:** Differentially expressed lnRNA in NPC microarray dataset identified by ncFANs reannotation.

Vega gene ID	LNCipedia gene ID	Locus conservation*	Exon number	Transcript size (bp)	Genome location	Adjust *P* value
OTTHUMG00000153695	lnc-BCL2L11-3	No	2	757	chr2:112248850-112268567/+	0.002
OTTHUMG00000002490	lnc-AL355149.1-1	No	2	342	chr1:16847189-16848303/+	0.004
OTTHUMG00000150697	lnc-C22orf32-1	No	2	544	chr22:42672361-42673057/+	0.006
OTTHUMG00000021416	lnc-ZNF674-1	No	2	626	chrX:46185359-46187080/−	0.030
OTTHUMG00000020561	lnc-TLR4-1	Mouse	6	1299	chr9:120410884-120419305/+	0.033

*Locus conservation in *Mus musculus* and *Danio rerio* compared to *Homo sapiens*.

**Table 3:** Association of lncRNA expression levels with the clinicopathological variables in primary undifferentiated NPC patients.

		lnc-ZNF674-1	lnc-AL355149.1-1	lnc-C22orf32-1
		*P* value		
Gender	Number			
Male	32	0.108	0.028*	0.033*
Female	10			
Age				
<49	19	0.733	0.970	0.810
>49	23			
T stage				
T1	11	0.030*	0.062	0.019*
T2–4	31			
Nodal status				
Negative	7	0.895	0.692	0.692
Positive	35			

*P value below 0.05 was considered statistically significant.
